# Archaeosomes and Gas Vesicles as Tools for Vaccine Development

**DOI:** 10.3389/fimmu.2021.746235

**Published:** 2021-09-10

**Authors:** Natalia Adamiak, Krzysztof T. Krawczyk, Camille Locht, Magdalena Kowalewicz-Kulbat

**Affiliations:** ^1^Department of Immunology and Infectious Biology, Institute of Microbiology, Biotechnology and Immunology, Faculty of Biology and Environmental Protection, University of Lodz, Lodz, Poland; ^2^Univ. Lille, CNRS, Inserm, CHU Lille, Institut Pasteur de Lille, U1019 – UMR9017 – CIIL – Center for Infection and Immunity of Lille, Lille, France

**Keywords:** archaeosomes, gas vesicles, GVNP, vaccines, Archaea

## Abstract

Archaea are prokaryotic organisms that were classified as a new domain in 1990. Archaeal cellular components and metabolites have found various applications in the pharmaceutical industry. Some archaeal lipids can be used to produce archaeosomes, a new family of liposomes that exhibit high stability to temperatures, pH and oxidative conditions. Additionally, archaeosomes can be efficient antigen carriers and adjuvants promoting humoral and cellular immune responses. Some archaea produce gas vesicles, which are nanoparticles released by the archaea that increase the buoyancy of the cells and facilitate an upward flotation in water columns. Purified gas vesicles display a great potential for bioengineering, due to their high stability, immunostimulatory properties and uptake across cell membranes. Both archaeosomes and archaeal gas vesicles are attractive tools for the development of novel drug and vaccine carriers to control various diseases. In this review we discuss the current knowledge on production, preparation methods and potential applications of archaeosomes and gas vesicles as carriers for vaccines. We give an overview of the traditional structures of these carriers and their modifications. A comparative analysis of both vaccine delivery systems, including their advantages and limitations of their use, is provided. Gas vesicle- and archaeosome-based vaccines may be powerful next-generation tools for the prevention and treatment of a wide variety of infectious and non-infectious diseases.

## Introduction

Archaea constitute the domain consisting of small, single-celled organisms lacking a cell nucleus. They are generally considered non-pathogenic to humans. Most known species inhabit extreme environments, such as underwater hydrothermal vents, glaciers or brine lakes. Therefore, they are called extremophiles and include thermophilic, psychrophilic, acidophilic, alkalophilic, piezophilic and halophilic microorganisms, as well as polyextremophilics, such as thermoacidophiles. Metabolites and cellular components of extremophilic archaea are thus resistant to extreme physical and chemical conditions. Hence, they are widely used in environmental protection (amidases, nitrilases), molecular biology studies (proteases, DNA polymerases) and industry, such as the food and feed, pharmaceutical (lipases, esterases, xynalases, chitinases, CGTases, pullulanases, nitrilases, glucosidases), paper (lipases, esterases, cellulases, endoglucanases, xynalases), detergent (lipases, esterases, proteases, α-amylases, CGTases, pullulanases, cellulases, nitrilases, glucosidases), chemical (lipases, esterases, proteases, nitrilases), agrochemical (lipases, esterases, nitrilases), cosmetic (canthaxanthin), holographic (bacteriorhodopsin), textile (cellulases, endoglucanases, glucosidases), biofuel (lipases, esterases, α-amylases, glucoamylases, pullulanases, amylopullulanases, cellulases) and medical industry (nitrilases, bacteriorhodopsin, bacterioruberin, diketopiperazines, polyhydroxyalkanoates, archaeocins, archaeosomes, gas vesicles) (1–5).

In the medical industry, archaeal enzymes, e.g. nitrile-degrading enzymes called nitrilases, are used for the production of drugs, such as Ibuprofen, Naproxen, Gabapentin, Pregabalin, Atorvastation and penicillin (1). Bacteriorhodopsin is used for the production of artificial retinas for patients with retinitis pigmentosa (2), while bacterioruberin (red pigment) is being tested for its anti-cancer effect. Bacterioruberin of 3 different halophilic species, *Halogeometricum limi* RO1-6, *Haloplanus vescus* RO5-8 and *Halobacterium halobium*, has shown cytotoxicity on hepatocellular adenoma HepG2 cells (3). Diketopiperazines, produced at industrial scale by halophilic *Naloterrigena hispanica* and *Natronococcus occultus*, present antimicrobial, antifungal, antiviral and antitumor activity. They also affect the blood clotting process and inhibit quorum sensing in *Pseudomonas aeruginosa* (2). Polyhydroxyalkanoates (PHAs) are polyesters produced by many types of halophilic archaea, such as *Haloferax mediterranei*, a potential industrial source of the compound. Due to the fact that PHAs are biodegradable and biocompatible with mammalian tissues and blood, they can be used as sutures, as well as dressing and osteosynthesis materials (4).

Archaeocins have also found potential use in the medical industry. They are antimicrobial proteins or peptides, including halocins and sulfolobiocin. Halocins are produced by halophilic archaea of the genera *Halobacterium*, *Halorubrum* and *Haloferax* (3). So far, 7 halocins have been described, but it is suspected that there are many more (6). Halocins proved to be active against other halophilic archaea and certain types of bacteria from hypersaline environments, such as *Halomonas*, *Rhadovibrio*, *Salisaeta* and *Pontobacillis* (3). Their effect on human pathogens has not been studied yet. Sulfolobiocin is produced by hyperthermophilic *Sulfolobus* species and shows activity against the genus *Sulfolobus*. Its effect on human pathogens also remains unexplored (6).

Some archaeal products may also serve as vaccine carriers of great potential. These include archaeosomes and gas vesicles, which can act both as transporters of vaccine antigens and as adjuvants. As purified and synthetic antigens are usually poorly immunogenic, they often require the addition of adjuvants and/or particulate carriers. Alum is the most widely used adjuvant/carrier and has been used for nearly a century. While alum strongly increases antibody responses to co-administered antigens, it has less stimulatory effects on T cell responses (7). More recently, additional adjuvants and vaccine carriers have been used in licensed vaccines, including oil-in-water emulsions, virosomes, outer membrane vesicles and liposomes, sometimes containing Toll-like-Receptor (TLR) agonists to enhance the activation of antigen-presenting cells (APC) (8). Archaeosomes (9) and gas vesicles from halophilic archaea (10) are relatively recent additions to this list and are the objects of this article.

## Archaeosomes

### Definition of Archaeosomes

Archaeosomes are liposomes consisting of at least one total polar lipid extract (TPL) from archaea. In contrast to eukaryotic ester-linked lipids, archaeal lipids are ether-bound (11). Archaeosomal membranes composed of ether-lipids can form bilayers, monolayers or a combination of both (12). The core of bilayer archaeosomes is composed of diether lipids called archaeols, while the core of monolayer archaeosomes contains tetraether lipids called caldarchaeols (12). Archaeols and caldarchaeols have irregularly branched, usually fully saturated phytanyl chains (20-40 carbon atoms long) connected by ether bonds to sn-2, 3 carbons of a glycerol backbone (12, 13).

The unique structure of archaeal lipids provides advantages of archaeosomes over traditional liposomes. They display higher stability to a wide range of pH (from pH as low as 2 for some archaeosomes to as high as 10.0), enhanced thermostability (from 4 to 65°C), low proton permeability and stronger immunostimulatory effects (12, 14, 15). Moreover, their structures include both hydrophilic and hydrophobic domains, allowing both hydrophobic and hydrophilic substances to be entrapped (12).

Natural archaeosomes consisting of non-modified archaeal TPLs are effective in delivering a wide range of antigens to APC for the initiation of strong specific humoral and cellular CD4^+^ and CD8^+^ immune responses, as well as cellular memory (12, 13, 15, 16). They are ideally suited as carrier systems and/or adjuvants, as they naturally target and are effectively phagocytosed by mononuclear cells, and can therefore effectively present antigens by APC. This facilitates the induction of humoral and cellular immune responses and long-term cellular memory (17). The induction of CD8^+^ cytotoxic T cell responses by archaeosomes *via* the stimulation of dendritic cells and MHC class I cross-presentation can be very effective against neoplastic cells and intracellular pathogens (12–14, 17). Natural archaeosomes are well tolerated when administered intramuscularly to animals (14) and strongly enhance antibody and T cell responses to entrapped antigens (18). Administration routes other than systemic routes have also been explored for archaeosomes, including transdermal, oral and nasal routes. Topical application of ovalbumin (OVA) included in archaeosomes resulted in strong antibody responses to OVA in mice with a Th2 bias (19). Similar results were obtained when influenza vaccine was used as antigen and administered topically in an archaeosome formulation, which also resulted in a strong IFN-γ response (20). A comparison of archaeosomes composed of TPLs from three different archaea applied topically revealed differences in their ability of antigen distribution and vesicle accumulation in the skin epidermis (21). An archaeosome formulation containing total antigen extract from *Leishmania braziliensis* topically administered to mice induced a stronger antibody response to the *L. braziliensis* antigens than a lipid formulation, most likely because of its improved stability and uptake by APC (22). Similarly, OVA formulated in archaeosome vesicles was more immunogenic when given orally than OVA formulated in liposomes, likely because of superior stability in gastric and intestinal fluids (23). Nasal administration of archaeosome-formulated antigens also induces strong systemic immune responses and induces IgA responses in the nasal cavity, in the bile and vagina when aggregated with calcium or combined with a mucosal adjuvant (24).

#### Semi-Synthetic Archaeosomes

Traditionally produced, natural archaeosomes are mixtures of glycolipids for which lot consistency and reproducibility of the formulations are difficult to control (16). To improve consistency of archaeosomes, simplified, semi-synthetic formulations have been developed, which consist of a single lipid called sulfated S-lactosylarchaeol (SLA), in which the sulfated saccharide group is covalently linked to the free sn-1 hydroxyl backbone of the archaeol core (14–16, 25). The chemical formula of SLA is 6’-sulfate-β-D-Galp-(1,4)-β-D-Glcp-(1,1)-archaeol (14, 15). SLA ensures consistency combined with an easy synthesis method and therefore reduced production cost (14).

Similar to traditional archaeosomes, SLA archaeosomes are capable of eliciting long-term cellular and humoral responses against various antigens and maintain a good safety profile (14–16, 26). They have been shown to be well tolerated without inducing adverse reactogenicity in mice at doses up to 10 times higher than those typically used in vaccines (14). Their adjuvant effect is comparable to or greater than that of widely used adjuvants, such as aluminum hydroxide, TLR3, TLR4 and TLR9 agonists, oil-in-water or water-in-oil formulations (11). SLA preparations cause intense local secretion of cytokines, retention of the antigen and immune cells at the vaccination site, and strong antigen uptake by APCs and other cells of the immune system, such as neutrophils and monocytes (14, 16). They are preferentially taken up by macrophages (16).

In addition to SLA archaeosomes, other semi-synthetic archaeosomes have also been tested in earlier studies as carriers and adjuvants for entrapped antigens (27). Semi-synthetic archaeosomes based on archaeol from *Halobacterium salinarum* to which various sugar moieties were grafted showed various types of immune responses to entrapped antigens. Immunization of mice with OVA entrapped into an archaetidylglycerophosphate-O-methyl archaeosome purified from *Haloferax volcanii* to which 45% gentiotriosylarchaeol, mannotriosylarchaeol or maltotriosylarchaeol were added resulted in enhanced CD8^+^ T cell and decreased antibody responses, compared to the same archaeosome formulation without the triglycosylarchaeols. In contrast, when all three triglycosylarchaeols were added to the archaeosome, the antibody response was less affected, while the enhanced CD8^+^ T cell response was still observed (28).

#### Encapsulated and Admixed Archaeosomes

Among the archaeosome formulations there are traditional encapsulated archaeosomes, in which the antigen is released from the interior of the carrier, and admixed archaeosomes, in which the antigens are not entrapped but mixed with empty archaeosomes (18). Due to the differences in antigen localization on admixed archaeosomes compared to encapsulated archaeosomes, it is hypothesized that admixed archaeosomes may induce alternative antigen presentation pathways to those induced by encapsulated archaeosomes (16). Despite these differences, both formulations have repeatedly been shown to elicit comparable humoral and cellular responses and to induce strong antigen and immune cell retention at the vaccination site, with strong local cytokine secretion (15, 16, 26, 29). Similarly, by comparing three different formulations, Jia et al. also found that entrapped and admixed archaeosomes induced similar antibody and T cell responses to the test antigens OVA and hepatitis B surface antigen (18). However, in a B16- OVA melanoma model, the encapsulated archaeosomes appeared to have a greater ability to activate CD8^+^ T cells by dendritic cells *in vitro* than admixed formulations, yet in an *in vivo* murine tumor model the activity of both formulations was comparable with respect to survival (16). On the other hand, in a Human papilloma virus-16 (HPV-16) model, admixed preparations containing plasmid DNA encoding L1, E6 and E7 of HPV-16 induced higher IFN-γ response than encapsulated archaeosomes, but showed comparable effects on tumor size and survival of the mice (13).

#### Combination of Archaeosomes With Adjuvants

Recent studies have shown that when OVA-containing SLA archaeosomes were co-administered with the TLR3 agonist poly(I:C), antibody responses to OVA were significantly enhanced after intramuscular administration over those obtained when OVA was formulated with SLA archaeosome or poly(I:C) alone (30). Antigen-specific cytotoxic T cell activity and IFN-γ production were also synergistically increased by the SLA archaeosome – poly(I:C) combination. A similar synergistic effect on antigen-specific cytotoxicity and IFN-γ production was also seen when the TLR9 agonist CpG instead of poly(I:C) was combined with the SLA archaeosomes. A synergistic effect of CpG or poly(I:C) with SLA archaeosomes could also be observed in a murine B16-OVA tumor model, in which the combination of SLA archaeosomes with poly(I:C) significantly delayed tumor growth and prolonged the mouse survival time compared to OVA formulations with SLA archaeosomes or poly(I:C) alone (31).

### Production of Archaeosomes

The first step in the production of archaeosomes is the isolation of lipids from methanogenic, halophilic or thermoacidophilic archaea species, like *Methanobrevibacter smithii*, *H. salinarum* or *Thermoplasma acidophilum* (12, 16, 17). However, the main source of lipids is *H. salinarum* (**Table 1**). The obtained lipid composition depends on the type of archaea. Archaeosomes made of archaeol mainly come from halophiles, while archaeosomes containing caldarchaeol are from acidophils, and archaeosomes composed of the mixture of these lipids are mainly from methanogens (12). The size of archaeosomes varies from 20-1,000 nm (12). The zeta potential is similar between different archaeosomal formulations and ranges from -52.6 to -77.7 mV with a negative surface charge, and the polydispersity index is in the range of 0.3 to 0.6, which is larger than that of liposomes, suggesting a broader particle size distribution (21).

**Table 1:** Archaeal vaccines.

Type of archaea	Producer	Vaccine carrier	Vaccine target	Antigen	Reference
**Halophilic**	*H. salinarum*	Admixed archaeosome	Melanoma B16-OVA	OVA	(29)
	*H. tebenquichense*	Encapsulated archaeosome	*T. cruzi*	*T. cruzi* homogenate	(32)
	*H. salinarum*	Encapsulated archaeosome	*L. monocytogenes*	Secretory proteins	(33)
	*H. salinarum*	Encapsulated archaeosome	*M. tuberculosis*	Secretory protein Rv3619c	(34)
	TPL from *H. salinarum*	Encapsulated and admixed archaeosome	HPV-16	pDNA coding L1, E6, E7	(13)
	SLA from modified *H. salinarum* lipids	Encapsulated SLA archaeosome	HBV	Surface antigen	(11)
	SLA from modified *H. salinarum* lipids	Encapsulated SLA archaeosome	Melanoma B16	TRP-2	(25)
	SLA from modified *H. salinarum*	Encapsulated and admixed SLA archaeosome	HCV	Heterodimer E1E2	(26)
	SLA from modified *H. salinarum* lipids	Encapsulated and admixed SLA archaeosome	Melanoma B16-OVA	OVA	(29)
	SLA	Admixed SLA archaeosome	*Schistosoma mansoni*	Cysteine protease SmCB	(35)
	*H. salinarum*	GVNP	SIV	Gag, Tat, Rev and Nef1 fragments	(36)
	*H. salinarum*	GVNP	*S. typhimurium*	SopB fragments	
	*H. salinarum*	GVNP	*C. trachomatis*	MOMP, OcmB and PompD fragments	
	*H. salinarum*	GVNP	*P. falciparum*	Enolase fragment	
**Methanogenic**	*M. smithii*	Encapsulated archaeosome	Melanoma B16	Gp-100, TRP-2	(37)
	*M. smithii*	Encapsulated archaeosome	Influenza virus	Hemagglutinin	(15)
	*M. smithii*	Encapsulated archaeosome	*V. cholerae*	Cholera toxin B subunit	(38)

The natural lipids of archaea needed for the production of archaeosomes can be obtained from total lipid extracts, which include polar lipids and neutral lipids (e.g. squalene). This can be done by extracting archaea biomass in a mixture of chloroform, methanol and water. In order to obtain a pure fraction of TPL, the extract can then be precipitated with acetone (12, 17).

In order to purify the obtained lipids, chromatography methods, such as column chromatography or preparative thin-layer chromatography are routinely used (12, 17). At this stage, lipids can be chemically modified by modifying the lipid head groups (12, 17). From natural or chemically modified lipids, archaeosomes carrying both hydrophilic and hydrophobic compounds can be produced by hydrating previously dried thin lipid layers (12, 15, 17).

Traditionally, encapsulated archaeosomes are produced by hydrating a thin layer of lipids in an antigen solution. As a result, a solution of archaeosomes with the entrapped antigen is formed (18). The archaeosome suspension can then be further processed to obtain a uniform size, e.g. by sonication, and can be separated from free antigen by centrifugation (13, 15–17).

However, due to the variable and relatively low encapsulation efficiency of the antigens, ranging from 5–40%, resulting not only in the loss of antigen, but also in increased cost of vaccine production and variable antigen concentrations in a vaccine, a new archaeosome formula was recently developed, referred to as admixed archaeosomes (29). They can be formed by admixing a soluble antigen solution into pre-formed, hollow archaeosomes, which provides a convenient, easy process of archaeosome formulations (13, 15, 29). Admixed archaeosomes do not result in loss of antigen during production and are highly reproducible, while maintaining strong adjuvant properties (16).

### Applications of Archaeosomal Vaccines

Several studies have shown that archaeosomal vaccines are capable of inducing immune responses against antigens. The effects of these carriers have been investigated with *Mycobacterium tuberculosis, Listeria monocytogenes*, *Vibrio cholerae*, *Trypanosoma cruzi, Schistosoma mansoni*, hepatitis B virus (HBV), Hepatitis C virus (HCV), HPV-16 and influenza virus, as well as with murine B16 melanoma cells.

#### Human Papilloma Virus-16

Karimi et al. (13) developed 2 vaccine formulations against HPV-16, which accounts for approximately 55-60% of all cervical cancer cases. The encapsulated and admixed formulations of the natural archaeosomes contained plasmid DNA encoding the capsid-building protein L1, as well as the E6 and E7 oncoproteins. Immunization of mice with either archaeosome-L1/E6/E7 induced humoral and cellular CD4^+^ and CD8^+^ responses, with high levels of IFN-γ, IL-2, IL-4 and IL-10. However, the admixed archaeosomes stimulated higher concentrations of these cytokines compared to the encapsulated archaeosomes. In addition, administration of archaeosomes to mice was associated with increased apoptosis of tumor cells, which reached 40 to 60% of the PBS controls, while the plasmid DNA alone led to 15 to 20% apoptosis (13). Empty archaeosomes were not tested for this readout. Furthermore, archaeosomes reduced and delayed the growth of tumors, as by day 28, the average tumor volume in mice immunized with archaeosomes-L1/E6/E7 was only 15% compared to that of the control mice. Recombinant plasmid DNA alone and empty archaeosomes led to a tumor size reduction of approximately 65% and 30%, respectively. Finally, while all mice in the control group had died by day 42, all mice immunized with archaeosomes were still alive at that time point. The recombinant plasmid alone and the empty archaeosome groups showed a slight delay in mortality compared to the PBS control (13).

#### Hepatitis C Virus

When admixed and encapsulated SLA archaeosomes containing the HCV H77 E1/E2 heterodimer were compared to other adjuvant formulations, the admixed vaccine provided the strongest humoral response (26). Moreover, the SLA-admixed formulation induced antibodies that strongly neutralized HCV pseudoparticles and prevented infection of Huh7.5 liver cells, whereas the encapsulated formulation did not. However, the encapsulated formulation provided the strongest CD4^+^ cellular response. Neither preparation induced CD8^+^ cell responses in this model (26).

#### Influenza Virus

When encapsulated and admixed SLA preparations, natural encapsulated archaeosomes, and the squalene-based adjuvant AddaVax, carrying the influenza virus haemagglutinin were compared to each other, all preparations were found to elicit humoral responses against the antigen (15). However, the encapsulated SLA archaeosomes and AddaVax-formulated haemagglutinin provided stronger protection against influenza virus than *M. smithii* archaeosomes and prevented weight loss in influenza virus-infected mice, which averaged 10% in the natural archaeosome vaccinated group. Maternal vaccination to protect pups was also assessed using SLA and AddaVax formulations. Six-week old female mice were vaccinated with either encapsulated or admixed archaeosomes once before pregnancy or once before and once during pregnancy. The anti-HA antibody titers in the blood of the pups were higher when the mothers had received two doses and were about 10 times lower than in the blood of the mothers. Pups born to mothers vaccinated with encapsulated SLA archaeosomes had lower titers than those that were born to mothers vaccinated with admixed SLA archaeosomes. After one week, the pups and mothers were infected with the influenza virus H1N1 strain PR8 and protection against the disease in pups and mothers was evaluated. Both immunization protocols with the admixed archaeosomes induced strong protection against virus-induced mortality in both mothers and pups, while a single maternal immunization with the encapsulated archaeosomes did not protect the pups (15). There appeared thus to be a correlation between protection against weight loss/death and antibody titers in the pups. These titers did not reach the threshold levels in the pups born to mothers vaccinated once with the encapsulated archaeosomes, while the mothers had antibody levels high enough to be protected.

#### Hepatitis B Virus

In order to evaluate the immunomodulatory properties of SLA archaeosomes and to compare them to other adjuvants, such as TLR3, TLR4 and TLR9 agonists, water-in-oil and oil-in-water emulsions, and aluminum hydroxide, a preparation containing encapsulated recombinant HBV surface antigen was produced. The formulation was found to induce strong humoral and CD8^+^ T cell responses and to stimulate the expression of IL-6, G-CSF, GM-CSF, KC, MCP-1 and MIP-2. The SLA archaeosomes showed strong adjuvant properties, similar to or stronger than other adjuvants and induced the strongest cytotoxic response among the tested formulations (11).

#### Melanoma B16

Four encapsulated natural archaeosomes containing separately Gp100 and TRP-2, a mixture of coentrapped melanoma antigens and a mixture of archaeosomal carriers of both proteins were reported to stimulate CD8^+^ T cell responses and to provide protection against cancer. Dipeptide vaccines were found to induce better and longer protection than monopeptide vaccines (37). Subsequently, it was shown that the TRP-2 protein encapsulated in the SLA archaeosome induced a strong CD8^+^ T cell response in mice and strong protection against B16 melanoma, comparable to that induced by the natural archaeosome formulation (25).

In the melanoma B16-OVA model, archaeosome vaccines against OVA and checkpoint inhibitor therapy consisting of anti-PD-1 and anti-CTLA-4 antibodies were combined. All formulations tested, i.e. admixed and entrapped SLA archaeosomes and encapsulated natural archaeosomes presenting OVA, enhanced the effects of checkpoint inhibition therapy by increasing the survival rate of mice and OVA-CD8^+^ T cell production and by reducing tumor growth in comparison to checkpoint inhibition therapy alone (29).

#### Trypanosoma cruzi

A vaccine formulation against *T. cruzi*, the parasite that causes Chagas disease, based on archaeosomes composed of *Halorubrum tebenquichense* lipids in which dissolved parasite homogenates had been encapsulated was found to be effective in stimulating specific humoral responses. In addition, immunized mice infected with *T. cruzi* trypomastigotes showed low blood parasite titers and high viability compared to the groups of unvaccinated mice (32).

#### Schistosoma mansoni

Perera et al. (35) prepared three vaccine formulations targeting Cathepsin B (SmCB), the most abundant cysteine protease in schistosomula and adult *S. mansoni* by combining the antigen with SLA archaeosomes or AddaWax™. Both vaccines provided 40% protection and reduced adult worm burden, liver and intestinal egg counts, with the SLA archaeosome formulation reaching 60.5% reduction and AddaWax 86.8%. Both formulations also induced humoral and cellular immune responses. The SLA archaeosome formation increased IL-3 and Th1 cytokine levels, while AddaWax stimulated Th17 and Th2 cytokine production.

#### Listeria monocytogenes

In a vaccine formulation against *L. monocytogenes*, secretory proteins obtained from the culture supernatant of this pathogen were encapsulated in archaeosomes composed of *H. salinarum* lipids. Vaccination with this formulation resulted in increased production of the Th1 cytokines IFN-γ and IL-12, induction of humoral and cytotoxic T cell responses with the generation of T cell memory, without inducing IL-4. One week after infection with *L. monocytogenes*, a significant reduction in bacterial burden in the liver and spleen was noticed in the vaccinated animals compared to the controls, and after further 4 weeks, all vaccinated mice, unlike the control group, were protected against death (33).

#### Mycobacterium tuberculosis

A vaccine formulation against *M. tuberculosis* based on natural archaeosomes encapsulated with the Rv3619c antigen was compared to the most commonly used vaccine, the Bacille Calmette-Guérin, and shown to provide stronger IFN-γ and IL-12 responses, as well as proliferation of Rv3619-specific T cells, higher concentrations of co-stimulatory markers, stronger effector memory T cell responses and significantly greater reduction in bacterial load in the lungs and spleen after *M. tuberculosis* challenge (34).

#### Vibrio cholerae

The immune responses against the cholera toxin subunit B were also compared between preparations using liposomal and archaeosomal carriers. Archaeosomal vaccines prepared from *M. smithii* lipids appeared to induce significantly higher humoral responses compared to liposomes and similar responses compared to Freund’s complete adjuvant. Moreover, archaeosomal vaccines only required two administrations to achieve maximum antibody titers in mice (38).

## Gas Vesicles

### Definition of Gas Vesicles

Gas vesicles are small, inert, empty, proteinaceous intracellular organelles, produced by various microbes, including cyanobacteria, proteobacteria and halophilic and methanogenic archaea (36, 39, 40). In microorganisms inhabiting the water environment, gas vesicles play a role in the buoyancy of the cell, allowing it to adapt to environmental changes and to maintain access to oxygen and light required for ATP synthesis and optimal microbial growth (39–41). However, the role of gas vesicles in microorganisms such as methanogenic archaea or soil bacteria still remains unknown (36).

In the first stages of their biosynthesis, the vesicles are biconical and subsequently evolve into a cylindrical shape (39, 40). Usually, gas vesicles are 0.045 - 0.2 µm wide and 0.1 - 2 µm long, but their exact size and shape are determined by environmental conditions, e.g. the higher the pressure, the narrower the gas vesicles (39). Gas vesicles consist only of proteins. The basic composition of the vesicle structure includes two proteins: small hydrophobic GvpA proteins (7-8 kDa) and large hydrophilic GvpC proteins (20-40 kDa) (39, 40).

The GvpA proteins are the main component of the gas vesicle, building its core. GvpA forms 4.6 nm-wide ribs that spiral up perpendicular to the long axis of the gas vesicle. The hydrophobic vesicle wall composed of a single layer of GvpA proteins is 2 nm thick and allows diffusion of gases in both directions (23). The GvpC proteins form a hydrophilic mesh on the outer surface of the gas vesicle, strengthening its stability and structure (39, 40), but do not play an important role in membrane integrity. The *H. volcanii* 1C mutant containing all *gvp* genes except for *gvpC* still produces gas vesicle structures, although they take an unusual shape (41).

The production of gas vesicles and its regulation are driven by the differential expression of *gvp* gene clusters and often depend on environmental parameters (24). Besides the *gvpA* and *gvpC* genes, there are many other genes with unknown functions. While there is homology between the major genes encoding GvpA and GvpC of different organisms, distinct variations exist for the rest of the genes (39). The *gvp* cluster encodes between 8-14 Gvp proteins, which are found both on chromosomes and plasmids (40). A total of 14 *gvp* genes were identified in the *Halobacterium* NRC-1 strain, organized into two divergent operons, *gvpACNO* and *gvpDEFGHIJKLM* (37, 39, 41). While the function of most proteins remains unclear, it has been proposed that they are less important structural proteins or perform regulatory functions (23). It is known that not all Gvp proteins are necessary for the formation of gas vesicles. GvpC, GvpD, GvpE, GvpH and GvpI are redundant (41). GvpD and GvpE are regulatory proteins. GvpD represses the expression of *gvpA* and *gvpC*, while GvpE activates their expression (40, 41). Among these genes the most widely used for biotechnological purposes is *gvpC*. Several studies have shown that foreign sequences inserted into *gvpC* in *Halobacterium* sp. NRC-1 allowed the effective presentation of antigens on the surface of the vesicle (39).

Gas vesicles were first proposed as antigen carriers almost 20 years ago. Since then, they have been studied for the presentation of viral, bacterial and eukaryotic antigens (39). For this purpose, gas vesicle nanoparticles (GVNPs), i.e. gas vesicles isolated from producer cells modified genetically to present the desired antigen, have been used (39). Most of these studies have been performed using GVNPs obtained from *Halobacterium* sp. NRC-1 (39).

GVNPs are in the size range of the nanometer and therefore are ideally suited as carriers of drugs and vaccines. They have been shown to induce both humoral and cellular responses, as well as cellular memory. The mechanisms by which GVNPs induce immune responses include the presentation of antigens by APC and the cross-presentation of vaccine antigens (42). Moreover, *Halobacterium* sp. NRC-1 GVNPs not containing foreign antigens have been shown to stimulate the immune response without the addition of an exogenous adjuvant, and can therefore function both as antigen carriers and as inbuilt adjuvant (39).

### Production of GVNP Vaccines

Genetic engineering techniques have been used to obtain GVNPs presenting various antigens. The codon-modified DNA sequence corresponding to the desired antigens can be inserted into the gene sequence corresponding to the acidic tail at the C-terminal moiety of GvpC, which makes up the outer surface of the gas vesicle. As a result, the antigen is expressed on the surface of the gas vesicle (39). Halophilic archaea that produce gas vesicles include: *H. salinarum*, *H. mediterranei* and *Haloquadratum walsbyi*, although *H. salinarum* is the leading producer of GVNPs (**Table 1**) (39). To obtain purified gas vesicles, the cells have to be lysed. For *Halobacterium* sp. NRC-1 the cells can be lysed simply by incubating the recombinant halophiles in water. The cell lysate can then be subjected to low-speed centrifugation overnight to harvest the gas vesicles floating on the surface of the supernatant (39, 41). Gas vesicles are stable in water or detergent solutions and only dissolve in 80% formic acid (41).

### Applications of GVNP Vaccines

Various antigens have already been presented on the surface of GVNPs, such as simian immunodeficiency virus (SIV) antigens, as well as antigens from *Salmonella enterica* serovar Typhimurium, *Chlamydia trachomatis* and *Plasmodium falciparum*.

#### Simian Immunodeficiency Virus

In the case of SIV, fragments of Gag, Tat, Rev and Nef1 were expressed on the surface of GVNPs. Recombinant GVNPs presenting fragments of Gag (17, 168 or 235 amino acids-long peptides) induced IgG production and long-term immune memory. Anti-Gag antibodies were detected 120 days after a booster injection (36, 39). The fragments of Tat (50 amino acids), Rev (81 amino acids) and Nef1 (214 amino acids) were also expressed on GVNP. The recombinant GVNPs induced immune responses at levels similar to those obtained by Gag-GVNPs (36). During immunization with recombinant Tat-GVNP, Rev-GVNP and Nef1-GVNP vaccines, IL-10, IL-12 and IL-18 were produced (36, 39). The strongest immune response was shown after immunization with the recombinant Tat-GVNP vaccine (39).

#### *Salmonella enterica serovar* Typhimurium

To stimulate immunity against *S. enterica* serovar Typhimurium, GVNPs containing 2 fragments of SopB (100 amino acid-long SopB4 and 165 amino acid-long SopB5) have been prepared. Mice primed with an attenuated vaccine against *S*. *typhimurium*, followed by boosting with 100 µg of SopB4-GVNP or SopB5-GVNP 7 and 14 days later, developed a strong and long-lasting immune response against SopB and increased levels of the pro-inflammatory cytokines IFN-γ, IL-2 and IL-9, as well as GM-CSF in the serum. One week after oral challenge with 10^7^ colony-forming units of virulent *Salmonella* the bacterial loads in mesenteric lymph nodes, liver and spleen were at least 2 orders of magnitude lower than in mice boosted with non-recombinant GVNP. CD4^+^ T cell levels in the spleen were increased 2- to 4-fold compared to non-recombinant GVNP-boosted controls. Moreover, mice boosted with SopB5-GVNP survived 3-5 days longer than those boosted with non-recombinant GVNP (36, 42).

#### Chlamydia trachomatis

For the expression of *C. trachomatis* antigens on the GVNP surface, protein fragments of the major outer membrane protein MOMP (48 and 69 amino acids), the outer membrane B complex OcmB (162 and 144 amino acids) and the outer membrane polymorphic protein PompD (173 and 222 amino acids) have been used (36, 39). Immunostaining confirmed that GVNPs presenting the *C. trachomatis* antigens were absorbed by human foreskin fibroblast cells, in which they were gradually disintegrated and finally exposed on the surface of the fibroblasts (36, 39). Recombinant GVNPs were shown to engage TLR-4 and TLR-5 and to stimulate the production of TNF-α, IL-1β, IL-6 and IL-12 (36, 42).

#### Plasmodium falciparum

The highly conserved 15 amino acid-long fragment of the *P. falciparum* enolase (14 out of the 15 residues are identical between the *P. falciparum* and the *Plasmodium yoelii* peptide) was also expressed on GVNPs. Mice immunized with the recombinant GNVPs and then challenged with the murine *Plasmodium yoelii* parasite showed a lower degree of parasitemia and a longer survival rate compared to mice immunized with non-recombinant GVNPs (39). As *P. falciparum* does not infect mice, *P. yoelli* is routinely used for studies in mouse models (43). Likewise, the *P. falciparum* circumsporozoite protein was successfully expressed on the GVNP surface. However, the induced immune responses to this antigen have not yet been assessed (36, 44).

## Advantages and Limitations of Archaeosome and Gas Vesicle Vaccine Applications

Compared to liposomes, archaeosomes are characterized by greater resistance to high and low pH and temperature extremes, stronger resistance to oxidative stress, chemical hydrolysis and bile salts, lower proton permeability and a stronger immunostimulatory effect (12–14, 16). The thermostability of archaeosomes enables their sterilization and ensures their stability even without a cold chain (12, 17). Studies have also demonstrated that they are well tolerated when injected intradermally, do not induce toxic and other adverse effects in vaccinated animals, which indicates a good safety profile (12–14).

However, work with natural archaeosomes revealed important limitations of this carrier, which are related to the various lipid compositions between lots, which cause issues in lot consistency. The lipid composition of archaea includes a wide range of natural lipids, which is specific for each cell and changes throughout the growth phases of the culture. This precludes homogeneity among vaccine batches (18, 28). However, this problem has been solved by the development of semi-synthetic SLA archaeosomes, which improves the consistency of the lipid composition of the vaccines, while providing a greater or similar adjuvant effect as compared to natural archaeosomes (14–16, 25, 29).

A second limitation is that the encapsulated archaeosome formulation has a low efficiency of entrapping antigens, ranging from 5-40%, which results in loss of antigen during production and thus increased production costs, as well as uneven antigen-lipid ratios in the vaccine batches (16, 29). The admixed formulation has been proposed as the solution to the variations in the amounts of encapsulated antigens. Admixed formulations result in less antigen loss than encapsulated archaeosomes, hence the production cost is lower and the antigen-lipid ratio and lot consistency are easier to control (29).

GVNPs also present great biological stability, including resistance to chemical and enzymatic degradation, as well as high thermal stability, as they can be stored for several months at room temperature and even at 50°C (36, 39, 43). In addition, they show high biocompatibility and induce strong systemic immunity after both subcutaneous and intraperitoneal administration, without causing adverse post-vaccination events and systemic or local toxic effects (36). Gas vesicles induce stronger immune responses than subunit vaccines and are generally safer than attenuated vaccines, which suggests their potential use for immune-compromised subjects (36, 39). As the development of GVNP-based vaccines requires the use of genetic engineering technologies, their technical limitations are directly linked to the limitations of genetic engineering applicable to archaea. Antigens suitable for GVNP-based vaccines are limited to protein and peptide antigens. Furthermore, if precise post-translational modifications of proteins or peptides, such as glycosylation, are critical for the induction of protective immunity, it will be challenging to generate them in GVNPs. Therefore, the GVNP technology may be most suited for bacterial protein antigens, and only to a limited extend for viral, eucaryotic and cancer antigens.

**Table 2** summarizes the features of both archaeal vaccine carriers. Archaeosomes are liposomes composed of ether lipids obtained from methanogenic, halophilic and thermophilic archaea. GVNPs are empty, protein organelles, not only produced by halophilic and methanogenic archaea, but also by bacteria. Studies on archaeal antigen carriers showed the great potential of halophilic archaea in vaccine development. This is especially the case for halophilic *H. salinarum* which is a leading producer of GVNPs and lipids used in archaeosome production (**Table 1**). Archaeosomes, unlike GVNPs, do not require genetic engineering technologies for vaccine production and provide possibilities for modifications, such as chemical modifications of the lipid components to obtain SLA archaeosomes. Archaeosomes allow for both the presentation of antigens on their surface and the release of antigens from their interior, whereas GVNPs allow for the presentation of antigens only on their surface. The cargos presented by archaeosomes include proteins and plasmid DNA, potentially even mRNA, while GVNPs present only fragments of proteins. However, both carriers have successfully displayed viral, bacterial, and eukaryotic antigens, although so far, only archaeosomes have been used to present tumor antigens. Furthermore, both carriers share the ability to effectively induce cellular and humoral immunity and an excellent safety profile in animal models. However, no studies have yet been conducted to compare the immunostimulatory capabilities of these two archaeal vaccine carriers in head-to-head evaluations. **Table 3** summarizes information regarding the route of vaccination and the size of archeosomes and gas vesicles in different disease models.

**Table 2:** Comparison of archaeosomes and gas vesicle characteristics.

	ARCHAEOSOMES	GVNPs
**Definition**	liposomes composed of ether lipids	empty, proteinaceous organella
**Producers**	methanogenic, halophilic and thermophilic archaea	cyanobacteria, proteobacteria, methanogenic and halophilic archaea
**Leading producer**	*Halobacterium salinarum*	*Halobacterium salinarum*
**Production process**	does not require genetic engineering methods	requires genetic engineering methods
**Semi-synthetic formulation**	+	–
**Antigen presentation**	released from archaeosome and presented on archaeosome surface	presented on GVNP surface
**Types of antigens**	proteins, long peptides, DNA	protein fragments
**Source of antigens**	viral, bacterial, eucaryotic	viral, bacterial, eucaryotic
**Tumor antigens presentation**	+	–
**Induced response**	humoral and cellular	humoral and cellular
**Safety of use**	+	+

**Table 3:** Particle size and the mode of delivery of the archaeosomes and gas vesicles in different disease models.

	Mode of delivery/route of vaccination	Size (diameter) [nm]	Disease model/antigen	Reference
**ARCHAEOSOMES**	subcutaneously intramuscularly subcutaneously subcutaneously subcutaneously subcutaneously subcutaneously intramuscularly subcutaneously intramuscularly intramuscularly intraperitoneally intramuscularly subcutaneously intraperitoneally subcutaneously intranasally transdermal	100-200 NS 159-271 564 99.15 NS 127-429 92-266 172-202 159-271 NS 63-207 130-220 50-100 100 150-300	melanoma influenza virus melanoma Chagas disease listeriosis tuberculosis cervical cancer hepatitis melanoma HCV schistosomiasis BSA, OVA, HEL cholera toxin B subunit listeriosis tularemia OVA	(18) (15) (29) (32) (33) (34) (13) (11) (25) (26) (35) (9) (38) (45) (46) (21)
**GVNPs**	intraperitoneally *in vitro* analysis *in vitro* analysis subcutaneously	NS 300 NS NS	salmonellosis malaria trachoma SIV	(36) (44) (47) (10)

NS, non-specified; BSA, Bovine Serum Albumin; OVA, ovalbumin; HEL, hen egg lysozyme; SIV, simian immunodeficiency virus.

The structure of archaeosomes offers the possibility of improving their formulation and adapting them to generate the desired type of immune responses, which is their strong advantage over GVNPs. This is reflected by the strong interest of investigators in the semi-synthetic SLA and admixed formulations of the archaeosomes. Recent studies have now engaged in comparisons of different archaeosomes aiming at determining the optimal formulations for a given antigen and comparing their immunostimulatory abilities to commonly used commercial adjuvants (16).

## Conclusion

Although both archaeosomes and GVNPs are able to effectively present exogenous antigens and induce strong cellular and humoral responses, as well as cellular memory, so far, all *in vivo* studies on archaeosomes and GVNPs have been conducted only in murine models. Immunogenicity, vaccine efficacy and safety studies in other animals, including humans, are lacking. It remains therefore be investigated whether the properties of GVNPs and archaeosomes observed in murine models can be extrapolated to vaccine-target animal species, including humans. If lot consistency issues can be solved in a satisfactory manner and acceptable safety and immunogenicity can be established, GVNP and archaeosome-based vaccines may be powerful next-generation tools for the prevention and treatment of a wide variety of infectious and non-infectious diseases.

While most investigations have carried out with the intramuscular, intraperitoneal or subcutaneous route of vaccination, alternative routes, such as transdermal or mucosal routes may be attractive alternatives, as they are needle free and may induce potent mucosal immunity in addition to systemic immunity. Several studies on these alternative routes have been performed with archaeosomes, but these routes have yet attracted limited attention for GVNPs, although one study has explored the transdermal route of vaccination with GVNPs (48). While GVNPs, like archaeosomes have in-built adjuvant properties, the addition of exogenous adjuvants that orient and further strengthen the desired immune responses have only recently started to be explored for archaeosomes, but have not yet been investigated with GVNPs. Together with targeting relevant species, other than mice, for given diseases that need to be assessed for the potency of archaeosomes and GVNPs, investigations of alternative vaccination routes and adjuvant combinations may reveal the true potential of these archaeal vaccine carriers and may help to generate interest of developers of vaccines for human or animal use.

## Author Contributions

NA and MK-K conceptualized the manuscript. NA and MK-K wrote the original draft. CL made substantial contributions to discussions of the content and revised the manuscript. KK contributed to design and table content and manuscript editing. All authors contributed to the article and approved the submitted version.

## Funding

This work was supported by the National Science Centre grant no 2017/27/N/NZ6/02850.

## Conflict of Interest

The authors declare that the research was conducted in the absence of any commercial or financial relationships that could be construed as a potential conflict of interest.

## Publisher’s Note

All claims expressed in this article are solely those of the authors and do not necessarily represent those of their affiliated organizations, or those of the publisher, the editors and the reviewers. Any product that may be evaluated in this article, or claim that may be made by its manufacturer, is not guaranteed or endorsed by the publisher.
